# Study on the Stability of Reference Genes and HSP60 for Expression Analysis in *Chilo suppressalis* in Response to Humidity Stress

**DOI:** 10.3390/insects17010072

**Published:** 2026-01-07

**Authors:** Ming Zhao, Yong Chen, Hai-Bo Zhang, Jian-Fei Mei, Ya-Jun Guo

**Affiliations:** 1 College of Plant Protection, Yangzhou University, Yangzhou 225009, China; clark.chen@yzjrd.com; 2 Jiangsu Plant Protection and Plant Quarantine Station, Nanjing 210036, China; zhanghbjs@126.com; 3 Guizhou Zhuohao Agricultural Science and Technology Co., Ltd., Zunyi 563100, China; mjf.cn@live.cn; 4 Agricultural Technology Comprehensive Service Center of Jiangdu District, Yangzhou 225200, China; jdgyj@163.com

**Keywords:** qRT-PCR analysis, reference gene, desiccation, rehydration, *Chilo suppressalis*

## Abstract

Quantitative real-time PCR (qRT-PCR) is a high-reliability, -sensitivity, and -operability technique to quantify gene expression. It is necessary to select stable reference genes for normalization. Water plays important roles in the metabolism, physiology, distribution, and so on, in insects. In this study, the suitability of various reference genes for qRT-PCR analysis was evaluated in different developmental stages of *Chilo suppressalis* exposed to desiccation or rehydration stress. The ∆Ct method, geNorm, NormFinder, and BestKeeper were used to evaluate the suitability of nine reference genes for normalizing gene expression in the third instar larvae, the fifth instar larvae, male pupae, female pupae, male adults, and female adults under different humidities. The results indicated that *18S rRNA* was the most stable reference gene for monitoring gene expression in the third instar larvae, while *ACTIN*, *TUB*, *UBI*, *UBI*, and *EF1* were the optimal genes for the fifth instar larvae, male pupae, female pupae, male adults, and female adults, respectively. The optimal number of reference genes recommended by geNorm analysis indicated that two candidate reference genes were sufficient for data normalization under all experimental conditions tested. To validate these recommendations, the expression profile of the gene encoding heat shock protein 60 (*Hsp60*) was investigated. *Hsp60* transcript levels showed significant differences when normalized to the most stable single reference gene, or combined reference genes, compared with the least stable reference gene. The reference genes identified in the present study will enhance the reliability of gene expression data for *C. suppressalis* under humidity stress.

## Simple Summary

Quantitative real-time PCR (qRT-PCR) is necessary to select stable reference genes for normalization. In this study, the suitability of various reference genes for qRT-PCR analysis was evaluated in different developmental stages of *Chilo suppressalis* exposed to desiccation or rehydration stress. The results indicated that *18S rRNA* was the most stable reference gene for monitoring gene expression in the third instar larvae; *ACTIN*, *TUB*, *UBI*, *UBI*, and *EF1* were the optimal genes for the fifth instar larvae, male pupae, female pupae, male adults, and female adults, respectively. The optimal number of reference genes recommended by geNorm analysis indicated that two candidate reference genes were sufficient for data normalization under all experimental conditions tested. To validate these recommendations, the expression profile of *Hsp60* was investigated. *Hsp60* transcript levels showed significant differences when normalized to the most stable single reference gene, or combined reference genes, compared with the least stable reference gene. The reference genes identified in the present study will enhance the reliability of gene expression data for *C. suppressalis* under humidity stress.

## 1. Introduction

Regulation of water balance is a primary component of homeostasis in all organisms. In insects, water also plays crucial roles in metabolite transport, maintenance of macromolecular structures, and determination of spatiotemporal distribution variations [1,2]. However, due to relatively small body size and high surface-to-volume ratio, insects face significant challenges in maintaining water balance [3,4]. Consequently, insects have evolved many strategies to manage water, such as an integument with abundant hydrophobic epicuticular lipids, acclimation treatments, and proteins that regulate water permeability [5,6]. With global climate change, environmental conditions including extreme temperatures, altered rainfall patterns, and droughts will occur frequently. It is very important to understand the responses of insects to water availability, because it has a pronounced influence on their survival, distribution patterns, and species richness [1,2]. Nevertheless, the molecular mechanisms underlying water rehydration and desiccation resistance in insects remain poorly understood [4,5].

The striped stem borer, *Chilo suppressalis* (Walker), is an important rice and water oat pest widely distributed in temperate and subtropical Asia, southern Europe, and North Africa. In China, the first instar to sixth instar larvae of *C. suppressalis* bore into the stems of rice and water oats, representing the main damaging stage of this pest to crops. The damage caused by *C. suppressalis* has spread to more than ten provinces in China and is gradually increasing [6]. The larvae prefer dry environments for pupation, while the pupae favor high-humidity conditions, but are intolerant of waterlogging. After a few days, the pupae emerge as adults, which lay eggs on host plants. All developmental stages of *C. suppressalis* favor high-humidity environments. In the control of *C. suppressalis*, humidity is often used to regulate its damage. To improve green control of *C. suppressalis*, clarifying the molecular mechanisms underlying its response to desiccation or rehydration stress at different developmental stages is crucial.

More accurate genomes and more transcriptomes of *C. suppressalis* are available [7,8]. Gene expression analyses are important for identifying the functions of different genes in various organisms. Quantitative real-time PCR (qRT-PCR) is a high-reliability, -sensitivity, and -operability technique, which is available to measure and evaluate functional gene expression [9,10]. Although qRT-PCR is often described as the gold standard for gene expression analysis, the reliability of results is directly affected by some factors, such as RNA quality and quantity, reverse transcription efficiency, and primer design [11,12]. Therefore, accurate gene expression normalization, using proper reference genes, is crucial to obtaining reliable gene expression levels. Reference genes, often called housekeeping genes, are based on their supposed constitutive expression, such as *ACTIN*, 18S ribosomal RNA (*18S rRNA*), and tubulin (*TUB*). However, it is noteworthy that these genes are influenced by different treatment conditions, and no universal reference gene exists [13,14]. In addition, the number of reference genes used for normalization can also affect the results of qRT-PCR [4,15]. Consequently, in order to ensure accurate results, it is crucial to validate the reference genes with respect to the specific experiment.

In this study, expression stability of nine candidate reference genes in different developmental stages of *C. suppressalis* under desiccation or rehydration was evaluated, including *18S rRNA*, *ACTIN*, *TUB*, elongation factor 1 (*EF1*), ubiquitin-conjugating enzyme (*UBI*), nicotinamide adenine dinucleotide dehydrogenase (*NADHD*), histone 3 (*H3*), arginine kinase (*AK*), and ribosomal protein S11 (*RPS11*). To further evaluate the results, the expression profile of heat shock protein 60 gene (*Hsp60*) was analyzed. The findings of this study can provide support for future research on genes related to water adaptation in this important pest species.

## 2. Materials and Methods

### 2.1. Insects

*C. suppressalis* were collected from the rice fields of Yangzhou (32.39° N, 119.42° E) and reared on an artificial food source in an environmental chamber at 28 ± 1 °C with a 16:8 (light/dark) photoperiod and 70 ± 5% relative humidity (RH).

### 2.2. Sample Collection Under Various Humidity Treatments

According to the preliminary experiment, the 5th instar larvae, male and female pupae, and male and female adults, were exposed to 25% (desiccation), 50%, 75%, and 95% (rehydration) relative humidities for 24 h in the humidity chamber with ±1.5% RH accuracy (SANTN HTC-100, Santeng Instrument Co., Ltd., Shanghai, China) at 27 °C. The 3rd instar larvae were exposed to 25% (desiccation), 50%, 75%, and 95% (rehydration) relative humidities for 12 h each. The thirty individuals of *C. suppressalis* were included in every treatment, and surviving *C. suppressalis* were frozen in liquid nitrogen and stored at −80 °C immediately.

### 2.3. Selection of Reference Genes

*ACTIN*, *TUB*, *EF1*, *H3*, *RPS11*, *NADHD*, *AK*, *18S rRNA*, and *UBI* were selected as reference genes, and the specificity and amplification efficiency of their primers were certificated (Table 1) [16].

### 2.4. RNA Isolation and Reverse Transcription

Total RNA was isolated from each sample using the SV Total RNA isolation system (Promega Z3100, Madison, WI, USA). RNA integrity was analyzed by agarose gel electrophoresis, and then RNA purity and concentration were measured by spectrophotometric measurements (Eppendorf Biophotometer plus, Hamburg, Germany). Then, 0.50 μg total RNA was used for cDNA synthesis using the Bio-Rad iScriptTM cDNA Synthesis Kit (Bio-Rad Laboratories, Berkeley, CA, USA).

### 2.5. Quantitative Real-Time PCR Analysis

cDNA with 1:10 dilution was used as template for qRT-PCR analysis. The PCR reaction mixtures contained 10 μL of iTaq Universal SYBR Green supermix (2×) (Bio-Rad), 1 μL of each gene-specific primer (10 μM), 2 μL of cDNA templates, and 6 μL ddH_2_O. All reactions were performed in CFX-96 real-time PCR system (Bio-Rad Laboratories, Berkeley, CA, USA). The specific procedure comprised 3 min of the initial denaturation at 95 °C, followed by 40 cycles of denaturation at 95 °C for 30 s, and 30 s at the Tm value of the primer pairs (Table 1) [16]. Melting curve analysis from 65 °C to 95 °C was performed to determine the specificity of the amplified PCR products. Every treatment included four replicates, and each replicate was assayed in three technical replicates.

### 2.6. Statistical Analyses

The threshold cycle (Ct) value is defined as the number of amplification cycles required for the fluorescent signal to reach a preset threshold, with this threshold set at a consistent fluorescence intensity across all reactions. The stability of the 9 candidate reference genes was evaluated using the ∆Ct method [17], BestKeeper [18], NormFinder version 0.953 [19], and GeNorm version 3.5 [20]. A ∆Ct method result with a lower standard deviation (SD) indicates a more stable gene. The BestKeeper creates an index, and lower index scores indicate greater stability. NormFinder estimates expression variation, in which lower values are more stable. The geNorm algorithm calculates an expression stability value (M) and compares the pair-wise variation (V) of a gene with others. The geNorm algorithm uses 0.15 as a cut-off, with a ratio of Vn/Vn + 1 exceeding 0.15 indicating the need for an additional reference gene [20].

### 2.7. Validation of Reference Genes

The *Hsp60* in *C. suppressalis* was selected as a target gene to validate stable reference genes. Relative expression levels of *Hsp60* in different treatments were conducted according to the 2^−∆∆Ct^ method [17]. *Hsp60* expression at 75% relative humidity was used as a control for calibration. For normalization using multiple reference genes, normalization was performed individually for each reference gene, followed by averaging the results. One-way analysis of variance (ANOVA) was performed to compare using SPSS v. 16.0.

## 3. Results

### 3.1. Total RNA Quality and PCR Amplification Specificity

The expression levels of *ACTIN*, *TUB*, *EF1*, *H3*, *RPS11*, *NADHD*, *AK*, *18S rRNA*, *UBI*, and *Hsp60* were investigated in different developmental stages of *C. suppressalis* under desiccation or rehydration by qRT-PCR (Table 2). The integrity, purity, and concentration of total RNA isolated from different samples were evaluated. The total RNA bands were intact on agarose gel electrophoresis, indicating that the RNA was of good integrity. The A260/280 ratios confirmed high purity for all RNA samples. Additionally, all ten genes produced amplicons that exhibited a single peak in melting curve analysis, demonstrating that each reaction amplified a unique product.

### 3.2. Expression Profiles of Reference Genes

**Figure 1:**
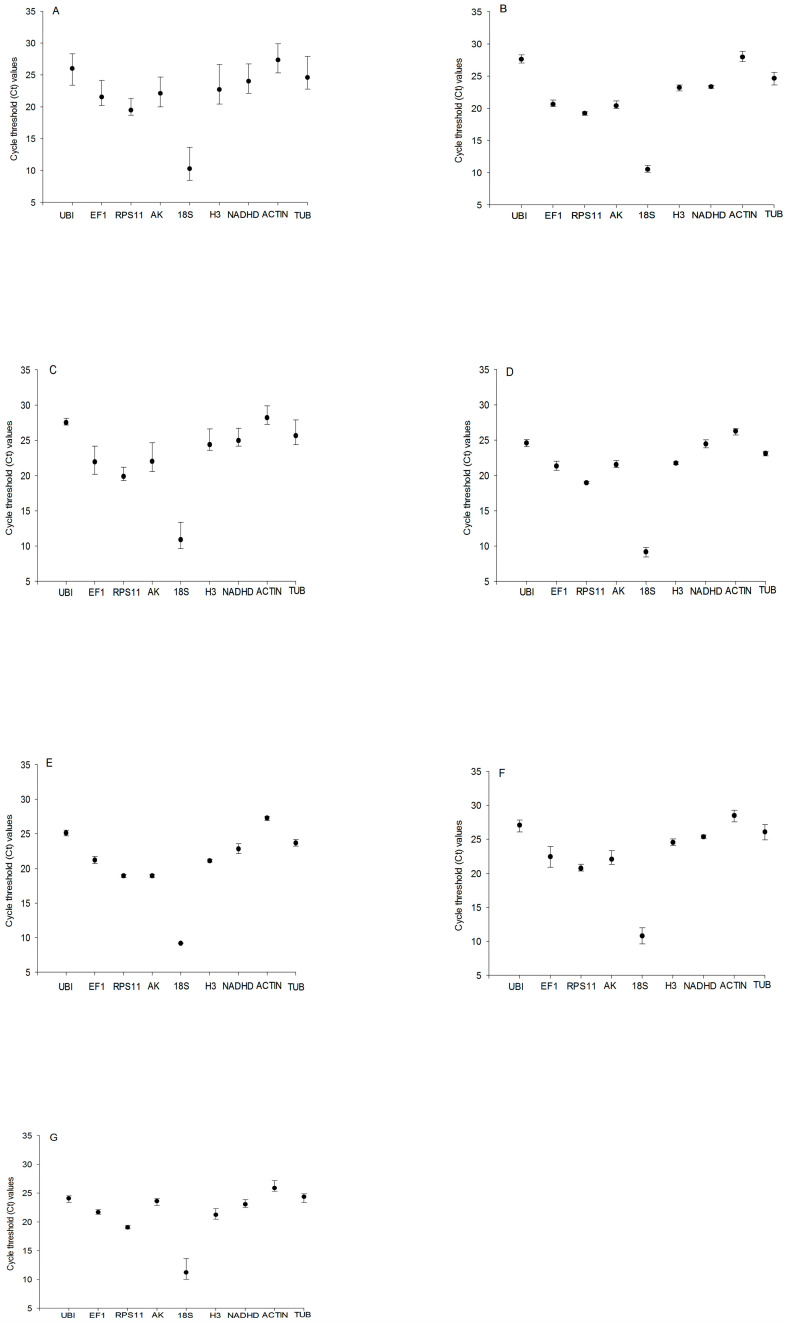
Expression levels of candidate reference genes in different developmental stages of *Chilo suppressalis* under desiccation or rehydration. Expression stability (Ct) of reference genes in the following samples: (**A**) all *Chilo suppressalis* samples (*n* = 113); (**B**) samples of the third instar larvae (*n* = 36); (**C**) samples of the fifth instar larvae (*n* = 36); (**D**) samples of one-day-old male pupae (*n* = 36); (**E**) samples of one-day-old female pupae (*n* = 36); (**F**) samples of one-day-old male adults (*n* = 36); and (**G**) samples of one-day-old female adults (*n* = 36). The black circles indicate the means of samples, and the bars indicate the minimum to maximum values.

Expression levels were presented as the number of cycles required for amplification to reach a fixed threshold (threshold cycle, Ct value). The t Ct values of the nine candidate reference genes under different experimental conditions were plotted. The mean Ct values of the nine reference genes ranged from 8.45 (*18S rRNA*) to 29.89 (*TUB*). *18S rRNA* (mean Ct 10.30) showed the most abundant expression levels, followed by *RPS11* (mean Ct 19.47), *EF1* (mean Ct 21.55), *AK* (mean Ct 22.12), *H3* (mean Ct 22.71), *NADHD* (mean Ct 24.01), *UBI* (mean Ct 24.60), *TUB* (mean Ct 26.00), and *ACTIN* (mean Ct 27.36) (Figure 1A). However, *RPS11* showed the smallest Ct variation (0.13), and *TUB* showed the biggest Ct variation (0.56) in samples of the third instar larvae (Figure 1B); for the fifth instar larvae, the smallest Ct variation (0.31) and the biggest Ct variation (1.32) were observed for *UBI* and *AK*, respectively (Figure 1C). *RPS11* showed the smallest Ct variation (0.10) and *EF1* showed the biggest Ct variation (0.52) in samples of the male pupae (Figure 1D), but, for the female pupae, the smallest Ct variation (0.03) and the biggest Ct variation (0.57) were observed for *18S rRNA* and *NADHD*, respectively (Figure 1E). *RPS11* showed the smallest Ct variation (0.15), and *EF1* showed the biggest Ct variation (1.22) in samples of the male adults (Figure 1F), but, for the female adults, the smallest Ct variation (0.24) and the biggest Ct variation (1.22) were observed for *RPS11* and *18S rRNA*, respectively (Figure 1G). These results indicated that no single reference gene was suitable for normalizing gene expression across all developmental stages of *C. suppressalis* under desiccation or rehydration.

### 3.3. Gene Analysis of Gene Expression Stability

For the third instar larvae, under desiccation or rehydration, four methods of comprehensive analysis found that *18S rRNA* and *NADHD* showed the highest expression stability. According to NormFinder, the stability ranking, from most to least stable, was *18S rRNA* > *EF1* > *H3* > *UBI* > *NADHD* > *RPS11* > *ACTIN* > *TUB* > *AK*. The comparative ∆Ct method, NormFinder, Best Keeper, and geNorm all revealed that *TUB* and *AK* were relatively unstable reference genes (Table 2). For the fifth instar larvae, under desiccation or rehydration, it was found that *ACTIN* and *TUB* showed the highest expression stability. According to NormFinder, the stability ranking, from most to least stable, was *ACTIN* > *TUB* > *H3* > *18S rRNA* > *NADHD* > *RPS11* > *AK* > *EF1* > *UBI*. NormFinder and geNorm both exhibited that *UBI* was the least stable reference gene (Table 2).

For the male pupae, under desiccation or rehydration, four methods of comprehensive analysis showed that *TUB* and *ACTIN* were most stable. According to NormFinder, the stability ranking, from most to least stable, was *TUB* > *ACTIN* > *UBI* > *AK* > *H3* > *RPS11* > *18S rRNA* > *EF1* > *NADHD*. NormFinder and geNorm both exhibited that *NADHD* was the least stable reference gene (Table 2). For the female pupae, under desiccation or rehydration, it was found that *TUB*, *ACTIN*, and *UBI* showed the highest expression stability. According to geNorm, the stability ranking, from most to least stable, was *UBI*/*ACTIN* > *TUB* > *AK* > *18S rRNA* > *EF1* > *H3* > *RPS11* > *NADHD*. The comparative ∆Ct method, NormFinder, Best Keeper, and geNorm all exhibited that *NADHD* was the least stable reference gene (Table 2).

For the male adults, under desiccation or rehydration, four methods of comprehensive analysis found that *UBI* and *H3* showed the highest expression stability. According to geNorm, the stability ranking, from most to least stable, was *RPS11*/*H3* > *NADHD* > *ACTIN* > *UBI* > *TUB* > *18S rRNA* > *AK* > *EF1*. The comparative ∆Ct method, NormFinder, Best Keeper, and geNorm all exhibited that *EF1* was the least stable reference gene (Table 2). For the female adults, under desiccation or rehydration, it was found that *EF1*, *RPS11*, and *UBI* showed the highest expression stability. NormFinder analysis revealed that the stability ranking (from most to least stable) was *EF1* > *NADHD* > *RPS11* > *H3* > *ACTIN* > *UBI* > *AK* > *TUB* > *18S rRNA*. The comparative ∆Ct method, NormFinder, Best Keeper, and geNorm all exhibited that *18S rRNA* was least stable reference gene (Table 2).

### 3.4. Optimal Number of Reference Genes

**Figure 2:**
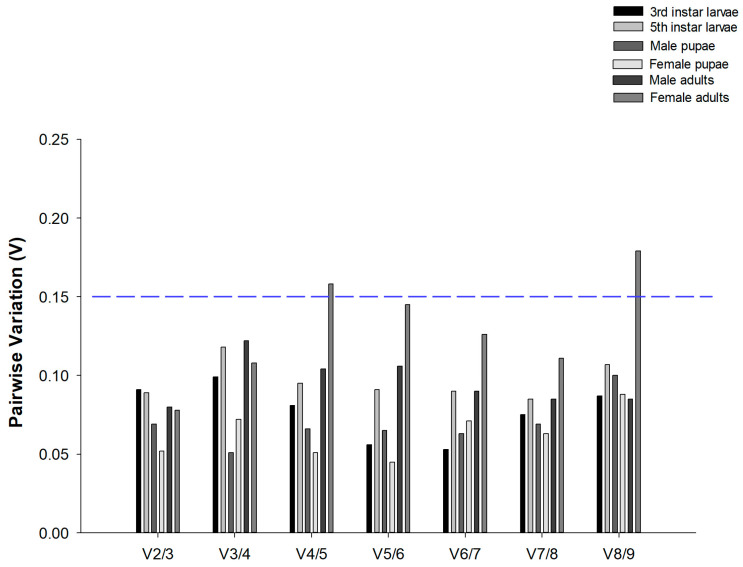
Optimal number of reference genes for normalization in *Chilo suppressalis*. The pairwise variation (Vn/Vn + 1) was analyzed by geNorm to determine the optimal number of reference genes. When values fell below blue dashed line at 0.15, additional genes were not necessary. In total, 42 data points were subjected to analysis.

geNorm analysis also ascertained the optimal number of reference genes required for data normalization. The results revealed that pairwise variation values of V_2/3_ were all below the 0.15 cut-off across all developmental stages of *C. suppressalis* under desiccation or rehydration (Figure 2). These results indicated that two candidate reference genes were sufficient for reliable data normalization.

### 3.5. Validation of Reference Genes with Hsp60

**Figure 3:**
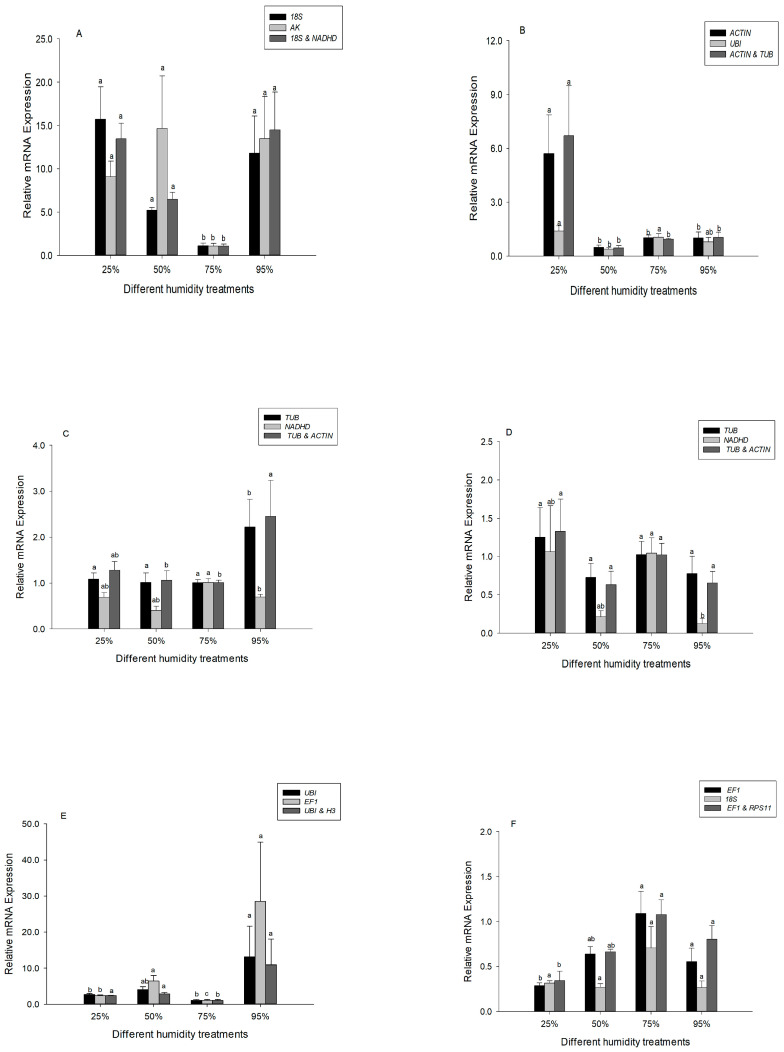
Expression levels of *Hsp60* in different developmental stages of *Chilo suppressalis* under desiccation or rehydration. (**A**) Expression of *Hsp60* in the third instar larvae was normalized with *18S rRNA* (most stable reference gene), AK (least stable), and two combined reference genes (*18S rRNA* and *NADHD*). (**B**) Expression of *Hsp60* in the fifth instar larvae was normalized with *ACTIN* (most stable reference gene), *UBI* (least stable), and two combined reference genes (*ACTIN* and *TUB*). (**C**) Expression of *Hsp60* in the male pupae was normalized with *TUB* (most stable reference gene), *NADHD* (least stable), and two combined reference genes (*TUB* and *ACTIN*). (**D**) Expression of *Hsp60* in the female pupae was normalized with *TUB* (most stable reference gene), *NADHD* (least stable), and two combined reference genes (*TUB* and *ACTIN*). (**E**) Expression of *Hsp60* in the male adults was normalized with *UBI* (most stable reference gene), *EF1* (least stable), and two combined reference genes (*UBI* and *H3*). (**F**) Expression of *Hsp60* in the female adults was normalized with *EF1* (most stable reference gene), *18S* (least stable), and two combined reference genes (*EF1* and *RPS11*). Values are means ± SE. Columns labeled with different letters indicate significant differences in expression (*p* < 0.05). At least 36 data points were used for analysis in each subfigure.

Heat shock protein 60 (*Hsp60*), a ubiquitous, highly abundant molecular chaperone, plays an important role in ecological adaptation. The relative expression of *Hsp60* was compared with different selected reference genes under desiccation or rehydration. For the analysis of the third instar larvae, we compared expression of *Hsp60* using the following selected reference genes: *18S rRNA* (most stable reference gene), *AK* (least stable), and the two recommended reference genes (*18S rRNA* and *NADHD*). The *Hsp60* expression profiles obtained using *18S rRNA*, *AK* and the combined reference genes resulted in a similar trend (Figure 3A). However, significant differences were detected when normalizing *Hsp60* expression in fifth instar larvae using *ACTIN* (most stable reference gene), *UBI* (least stable), and the combined reference genes (Figure 3B).

For the analysis of male and female pupae, we compared expression of *Hsp60* using the following selected reference genes: *TUB* (most stable reference gene), *NADHD* (least stable)*,* and the two recommended reference genes (*TUB* and *ACTIN*). The *Hsp60* expression profiles were significantly different between *TUB* or the combined reference genes and *NADHD* in male pupae (Figure 3C). In the female pupae, *Hsp60* expression was not induced when *TUB* (most stable reference gene) or the two recommended reference genes (*TUB* and *ACTIN*) were used to normalize expression. However, when *NADHD* (least stable) was used for normalization, the expression profile of *Hsp60* changed significantly (*F*_3, 8_ = 5.923, *p* = 0.020) (Figure 3D).

For the analysis of male adults, we compared expression of *Hsp60* using the following selected reference genes: *UBI* (most stable reference gene), *EF1* (least stable)*,* and the two recommended reference genes (*UBI* and *H3*). The *Hsp60* expression profiles were significantly different between the combined reference genes and *EF1* (least stable) (Figure 3E). In the female adults, *Hsp60* expression was not inhibited with *18S rRNA* (least stable) (*F*_3, 7_ = 2.901, *p* = 0.111). However, when the two recommended reference genes (*EF1* and *RPS11*) were used for normalization, the expression profile of *Hsp60* was inhibited significantly (*F*_3, 9_ = 7.932, *p* = 0.007) (Figure 3F).

## 4. Discussion

qRT-PCR has become an important technique for examining gene expression levels, due to its specificity, accuracy, and reproducibility. However, the accuracy of qRT-PCR requires robust normalization with reference genes. Extensive studies indicate that there is not a single ‘universal’ reference gene across all experimental circumstances [21,22,23]. In the current study, the expression levels of nine reference genes (*ACTIN*, *EF1*, *H3*, *18S rRNA*, *AK*, *NADHD*, *TUB*, *RPS11*, and *UBI*) were investigated in different developmental stages of *C. suppressalis* under desiccation or rehydration. These reference genes belong to different functional classes of proteins, to reduce the risk of co-regulation. Our results showed that the ∆Ct method, NormFinder, BestKeeper, and GeNorm exhibited distinct stability rankings for nine reference genes under desiccation or rehydration. Despite their differences, the four methods’ analyses resulted in similar rankings for the most and least stable reference genes under different experimental conditions; for instance, *NADHD*, *EF1*, and *18S rRNA* were ranked as the least stable genes in female pupae, male adults, and female adults, exposed to desiccation or rehydration, respectively. However, most reference genes identified by the four methods of analysis were different (Table 2). Similarly, there were discrepancies in stability rankings among the different programs in previous studies, which may be attributed to distinct logical algorithms [12,23]. Therefore, in this study, a comprehensive analysis of the stability rankings of the reference genes was further used.

*18S rRNA* and *ACTIN*, involved in basic cellular processes, are most commonly used reference genes for normalizing expression. Nevertheless, some studies have demonstrated that *18S rRNA* and *ACTIN* were not always reliable for normalizing gene expression [24,25]. In the present study, *18S rRNA* and *ACTIN* found in the third instar larvae, as well as male or female pupae, exposed to desiccation or rehydration, are appropriate reference genes. Consistent results have been reported in other studies [26,27]. *TUB* plays an essential role in maintaining cell shape, regulating cell division, and facilitating substance transport. *RPS11* plays an essential role in ribosomal assembly and protein translation. *EF1* functions in protein translation elongation by catalyzing GTP-dependent binding of aminoacyl-tRNA. *UBI* is critical for protein degradation and cell cycle regulation. These genes have been increasingly used as reference genes in various insect species [12,15,28,29]. Our results also suggested that *TUB*, *RPS11*, *EF1*, and *UBI* displayed high levels of stability under different experimental conditions. The combinations of the above reference genes were the best sets of grouped genes for normalizing expression in *C. suppressalis* under desiccation or rehydration. To further validate the reference genes, the expression patterns of *Hsp60* in different developmental stages of *C. suppressalis* exposed to desiccation or rehydration were analyzed. In general, the results demonstrated that the normalized expression levels of Hsp60, using the most stable reference genes or their combinations, were significantly different from those normalized using the least stable reference genes.

## 5. Conclusions

Insects have evolved various adaptive ecological strategies and molecular mechanisms to cope with humidity stress. However, the molecular mechanisms underlying insect adaptation to desiccation or rehydration remain poorly understood. Our study systematically investigated and validated nine potential reference genes for normalizing qRT-PCR data in *C. suppressalis* under desiccation or rehydration. Our findings showed that the expression stability of reference genes in *C. suppressalis* varied with developmental stages and sexes under humidity stress. To the best of our knowledge, this is the first study to validate reference genes in insects under desiccation or rehydration. These findings will facilitate future research on the molecular mechanisms of humidity stress adaptation in *C. suppressalis*.

## Figures and Tables

**Table 1:** Primer sequences and amplicon characteristics of the reference genes and *Hsp60* from *Chilo suppressalis*.

Gene	Accession No.	Primer Sequence (5′-3′)	Size (bp)	Tm (°C)	E (%)	R^2^
*ACTIN*	CSUOGS109568	AAAGAAACAGCAAAAGTCGGGG	243	56.0	93.2	0.977
		GTTCAATGGAGGTTCGGTAAGTAAA				
*18S*	GQ265912	GTGATGGGACGAGTGCTTTTATT	258	62.5	97.5	0.995
		GCTGCCTTCCTTGGATGTGG				
*H3*	CSUOGS102615	CTGCACCAAGCACTGGTGGA	184	56.0	107.5	0.993
		TAGCGGCGGACTGGAAACG				
*EF1*	CSUOGS104451	AAAATGGACTCGACTGAACCCC	137	56.6	94.0	0.994
		TCTCCGTGCCAACCAGAAATA				
*UBI*	CSUOGS107899	TCACCGACAGCAAACCAGACT	219	60.2	108.9	0.970
		GGAAGAAAACACCCCCCTCATATA				
*RPS11*	CSUOGS100188	ACTACCTTCACTACCTGCCCAAA	129	58.9	100.7	0.992
		ACGGCCTGCATTCTCCAAT				
*AK*	KT625596	GTTCTTGACCTTCTGCCCCAC	103	62.1	103.5	0.989
		GACTTCCTCCAGCTTAGCCTTGT				
*NADHD*	CSUOGS108441	GTACCATCAGCAGTTCCGTCG	264	60.1	107.6	0.995
		TCACCATAAGCACCTAAATCACCA				
*TUB*	EU429675	GAGGGCATGGACGAGATGGA	178	60.4	103.0	0.995
		ACGACGGTACGAGTATGACGGG				
*HSP60*	GQ265913	TGGCATTGACGGCTCTGTG	150	61.5	105.4	0.990
		TGGCATCTGTAAGTGCTGTTCG				

Note: The data have been certificated [16].

**Table 2:** Ranking order of reference genes of *Chilo suppressalis* under different experimental conditions.

Instar Stage	Rank	Delta Ct		Best Keeper		NormFinder		geNorm	
		Gene Name	Standard Deviation	Gene Name	Standard Deviation	Gene Name	Stability Value	Gene Name	Stability Value
**The third instar larvae**	1	18S	0.488	NADHD	0.13	18S	0.115	RPS11/ NADHD	0.144
	2	UBI	0.497	RPS11	0.19	EF1	0.128		
	3	H3	0.532	H3	0.27	H3	0.191	H3	0.236
	4	NADHD	0.537	EF1	0.33	UBI	0.193	18S	0.326
	5	RPS11	0.555	AK	0.37	NADHD	0.215	UBI	0.363
	6	EF1	0.584	18S	0.42	RPS11	0.246	EF1	0.380
	7	ACTIN	0.593	ACTIN	0.45	ACTIN	0.300	ACTIN	0.397
	8	AK	0.725	UBI	0.50	TUB	0.401	TUB	0.458
	9	TUB	0.729	TUB	0.56	AK	0.534	AK	0.541
**The fifth instar larvae**	1	ACTIN	0.625	UBI	0.31	ACTIN	0.143	AK/18S	0.158
	2	TUB	0.659	RPS11	0.65	TUB	0.215		
	3	RPS11	0.695	ACTIN	0.84	H3	0.243	TUB	0.234
	4	18S	0.749	NADHD	0.86	18S	0.283	H3	0.361
	5	H3	0.799	H3	1.11	NADHD	0.292	ACTIN	0.426
	6	UBI	0.807	TUB	1.12	RPS11	0.368	EF1	0.484
	7	AK	0.819	EF1	1.17	AK	0.396	NADHD	0.538
	8	EF1	0.849	18S	1.24	EF1	0.456	RPS11	0.586
	9	NADHD	1.032	AK	1.32	UBI	0.660	UBI	0.681
**Male pupae**	1	H3	0.516	RPS11	0.10	TUB	0.056	UBI/ ACTIN	0.112
	2	ACTIN	0.518	H3	0.15	ACTIN	0.115		
	3	RPS11	0.585	TUB	0.26	UBI	0.142	TUB	0.147
	4	TUB	0.589	ACTIN	0.28	AK	0.181	18S	0.222
	5	EF1	0.591	UBI	0.30	H3	0.219	AK	0.250
	6	18S	0.600	AK	0.34	RPS11	0.262	EF1	0.272
	7	AK	0.844	NADHD	0.36	18S	0.279	H3	0.341
	8	NADHD	1.163	18S	0.46	EF1	0.303	RPS11	0.390
	9	H3	0.516	EF1	0.52	NADHD	0.542	NADHD	0.483
**Female pupae**	1	TUB	0.394	18S	0.03	H3	0.078	UBI/ ACTIN	0.112
	2	ACTIN	0.414	RPS11	0.16	18S	0.101		
	3	UBI	0.454	H3	0.17	UBI	0.104	TUB	0.147
	4	18S	0.486	ACTIN	0.18	ACTIN	0.109	AK	0.222
	5	EF1	0.490	UBI	0.21	RPS11	0.187	18S	0.250
	6	H3	0.497	TUB	0.34	TUB	0.219	EF1	0.272
	7	AK	0.507	EF1	0.44	EF1	0.321	H3	0.341
	8	RPS11	0.543	AK	0.52	AK	0.435	RPS11	0.390
	9	NADHD	0.752	NADHD	0.57	NADHD	0.619	NADHD	0.483
**Male adults**	1	UBI	0.515	NADHD	0.14	UBI	0.080	RPS11/ H3	0.208
	2	ACTIN	0.546	H3	0.29	ACTIN	0.149		
	3	TUB	0.571	RPS11	0.30	TUB	0.231	NADHD	0.243
	4	H3	0.608	ACTIN	0.58	H3	0.338	ACTIN	0.370
	5	18S	0.672	UBI	0.70	RPS11	0.358	UBI	0.441
	6	RPS11	0.685	AK	0.75	AK	0.378	TUB	0.516
	7	NADHD	0.700	TUB	0.95	18S	0.393	18S	0.574
	8	AK	0.706	18S	1.10	NADHD	0.478	AK	0.618
	9	EF1	0.731	EF1	1.22	EF1	0.515	EF1	0.663
**Female adults**	1	UBI	0.429	RPS11	0.20	EF1	0.107	AK/TUB	0.144
	2	AK	0.456	EF1	0.28	NADHD	0.170		
	3	ACTIN	0.571	UBI	0.38	RPS11	0.295	UBI	0.211
	4	TUB	0.572	AK	0.39	H3	0.338	RPS11	0.325
	5	EF1	0.604	NADHD	0.42	ACTIN	0.429	EF1	0.517
	6	H3	0.632	TUB	0.49	UBI	0.531	NADHD	0.639
	7	NADHD	0.739	H3	0.53	AK	0.580	H3	0.712
	8	RPS11	0.871	ACTIN	0.64	TUB	0.684	ACTIN	0.763
	9	18S	1.227	18S	1.22	18S	1.106	18S	0.955

## Data Availability

The original contributions presented in this study are included in the article. Further inquiries can be directed to the corresponding author.

## References

[1] Chown S.L., Sørensen J.G., Terblanche J.S. (2011). Water loss in insects: An environmental change perspective. J. Insect Physiol..

[2] Knutelski S., Haranczyk H., Nowak P., Wróbel A., Leszczynski B., Okuda T., Strzalka K., Baran E. (2022). Rehydration of the sleeping chironomid, *Polypedilum vanderplanki* Hinton, 1951 larvae from cryptobiotic state up to full physiological hydration (Diptera: Chironomidae). Sci. Rep..

[3] Kühsel S., Brückner A., Schmelzle S., Heethoff M., Blüthgen N. (2017). Surface area-volume ratios in insects. Insect Sci..

[4] Wang Z.A., Receveur J.P., Pu J., Cong H.S., Richards C., Liang M.X., Chung H.Y. (2022). Desiccation resistance differences in *Drosophila* species can be largely explained by variations in cuticular hydrocarbons. eLife.

[5] Chown S.L., Janion-Scheepers C., Marshall A., Aitkenhead I.J., Hallas R., Liu W.P.A., Phillips L.M. (2023). Indigenous and introduced Collembola differ in desiccation resistance but not its plasticity in response to temperature. Curr. Res. Insect Sci..

[6] Lu M.X., He F.J., Xu J., Liu Y., Wang G.R., Du Y.Z. (2021). Identification and physiological function of *CsPrip*, a new aquaporin in *Chilo suppressalis*. Int. J. Biol. Macromol..

[7] Yin C., Liu Y., Liu J., Xiao H., Huang S., Lin Y., Han Z., Li F. (2014). *ChiloDB*: A genomic and transcriptome database for an important rice insect pest *Chilo suppressalis*. Database.

[8] Ma W.H., Zhao X.X., Yin C.L., Jiang F., Du X.Y., Chen T.Y., Zhang Q.H., Lin Q., Xu H.X., Hull J.J. (2020). A chromosome-level genome assembly reveals the genetic basis of cold tolerance in a notorious rice insect pest, *Chilo suppressalis*. Mol. Ecol. Resour..

[9] Huggett J., Dheda K., Bustin S., Zumla A. (2005). Real-time RT-PCR normalisation; strategies and considerations. Genes Immun..

[10] Bustin S.A., Beaulieu J.F., Huggett J., Jaggi R., Kibenge F.S., Olsvik P.A., Penning L.C., Toegel S. (2010). MIQE prècis: Practical implementation of minimum standard guidelines for fluorescence-based quantitative real-time PCR experiments. BMC Mol. Biol..

[11] Bustin S.A., Benes V., Garson J., Hellemans J., Huggett J., Kubista M., Mueller R., Nolan T., Pfaffl M.W., Shipley G.L. (2013). The need for transparency and good practices in the qPCR literature. Nat. Methods.

[12] Wang Z.B., Zhang H.F., Zhang Z.Y., Zhao J.Y., Ma F.L., Zheng M.M., Yang M.S., Sang X.Y., Ma K.S., Li L.L. (2022). Selection of reference genes for normalization of qRT–PCR analysis in the soybean aphid *Aphis glycines* Matsumura (Hemiptera: Aphididae). J. Econ. Entomol..

[13] Zhang S., An S., Li Z., Wu F., Yang Q., Liu Y., Cao J., Zhang H., Zhang Q., Liu X. (2015). Identification and validation of reference genes for normalization of gene expression analysis using qRT-PCR in *Helicoverpa armigera* (Lepidoptera: Noctuidae). Gene.

[14] Xu J., Welker D.L., James R.R. (2021). Variation in expression of reference genes across life stages of a bee, *Megachile rotundata*. Insects.

[15] Zheng Y.T., Li H.B., Lu M.X., Du Y.Z. (2014). Evaluation and validation of reference genes for qRT-PCR normalization in *Frankliniella occidentalis* (Thysanoptera: Thripidae). PLoS ONE.

[16] Xu J., Lu M.X., Cui Y.D., Du Y.Z. (2017). Selection and evaluation of reference genes for expression analysis using qRT-PCR in *Chilo suppressalis* (Lepidoptera: Pyralidae). J. Econ. Entomol..

[17] Schmittgen T.D., Livak K.J. (2008). Analyzing real-time PCR data by the comparative CT method. Nat. Protoc..

[18] Pfaffl M.W., Tichopad A., Prgomet C., Neuvians T.P. (2004). Determination of stable housekeeping genes, differentially regulated target genes and sample integrity: BestKeeper—Excel-based tool using pairwise correlations. Biotechnol. Lett..

[19] Andersen C.L., Jensen J.L., Ørntoft T.F. (2004). Normalization of real-time quantitative reverse transcription-PCR data: A model-based variance estimation approach to identify genes suited for normalization, applied to bladder and colon cancer data sets. Cancer Res..

[20] Vandesompele J., De Preter K., Pattyn F., Poppe B., Van Roy N., De Paepe A., Speleman F. (2002). Accurate normalization of real-time quantitative RT-PCR data by geometric averaging of multiple internal control genes. Genome Biol..

[21] Li H.B., Dai C.G., Zhang C.R., He Y.F., Ran H.Y., Chen S.H. (2018). Screening potential reference genes for quantitative real-time PCR analysis in the oriental armyworm, *Mythimna separata*. PLoS ONE.

[22] Kong D., Shi D., Wang C., Zhai R., Lyu L., He Y., Wang D. (2022). Identification and validation of reference genes for expression analysis using qRT-PCR in Cimex hemipterus (Hemiptera: Cimicidae). Insects.

[23] Yang Q.P., Li Z., Cao J.J., Zhang S.D., Zhang H.J., Wu X.Y., Zhang Q.W., Liu X.X. (2014). Selection and assessment of reference genes for quantitative PCR normalization in migratory locust *Locusta migratoria* (Orthoptera: Acrididae). PLoS ONE.

[24] Shang F., Wei D.D., Jiang X.Z., Wei D., Shen G.M., Feng Y.C., Li T., Wang J.J. (2015). Reference gene validation for quantitative PCR under various biotic and abiotic stress conditions in *Toxoptera citricida* (Hemiptera, Aphidiae). J. Econ. Entomol..

[25] Huang X.N., Gao Y.C., Jiang B., Zhou Z.C., Zhan A.B. (2016). Reference gene selection for quantitative gene expression studies during biological invasions: A test on multiple genes and tissues in a model ascidian *Ciona savignyi*. Gene.

[26] Teng X.L., Zhang Z., He G.L., Yang L.W., Li F. (2012). Validation of reference genes for quantitative expression analysis by real-time RT-PCR in four lepidopteran insects. J. Insect Sci..

[27] Paim R.M., Pereira M.H., Ponzio R.D., Rodrigues J.O., Guarneri A.A., Gontijo F.N., Araújo R.N. (2012). Validation of reference genes for expression analysis in the salivary gland and the intestine of *Rhodnius prolixus* (Hemiptera, Reduviidae) under different experimental conditions by quantitative real-time PCR. BMC Res. Note.

[28] Sun M., Lu M.X., Tang X.T., Du Y.Z. (2015). Exploring valid reference genes for quantitative real-time PCR analysis in *Sesamia inferens* (Lepidoptera: Noctuidae). PLoS ONE.

[29] Yang A.P., Wang Y.S., Huang C., Lv Z.C., Liu W.X., Bi S.Y., Wan F.H., Wu Q., Zhang G.F. (2021). Screening potential reference genes in *Tuta absoluta* with Real-time quantitative PCR analysis under different experimental Conditions. Genes.

